# Systemic Immuno-metabolic alterations in chronic obstructive pulmonary disease (COPD)

**DOI:** 10.1186/s12931-019-1139-2

**Published:** 2019-07-30

**Authors:** Amit R Agarwal, Smita Kadam, Ankita Brahme, Manas Agrawal, Komalkirti Apte, Govinda Narke, Kushal Kekan, Sapna Madas, Sundeep Salvi

**Affiliations:** Molecular Respiratory Research Laboratory, Chest Research Foundation, Sr. No 15, Marigold Premises, Behind Gold Adlabs, Pune, Pune, 411014 Maharashtra India

**Keywords:** Cigarette smoke, PBMCs, Glucose, Fatty acids, Metabolism

## Abstract

**Background:**

Metabolic adaptation in immune cells is necessary to modulate immune cell function as it is intricately coupled with intracellular metabolism. We aimed to characterize the metabolic state of human peripheral blood mononuclear cells (PBMCs) after long-term exposure to tobacco smoke in smokers with preserved lung function and COPD subjects.

**Methods:**

PBMCs were isolated from healthy non-smokers (HNS), healthy smokers (HS) and COPD subjects, cultured and the mitochondrial respiration while utilizing glucose (glycolysis), fatty acids (β-oxidation) or pyruvate (direct Krebs’ cycle substrate) was measured using the XFp Extracellular Flux Analyzer. Plasma levels of inflammatory cytokines IFN-γ, IL-17, TNF-α, IL-5, IL-9 and IFN-α were measured using flow cytometry. RAW264.7 cells were exposed to cigarette smoke condensate (CSC) for 1 h and its effect on cell viability, cellular metabolism and phagocytosis ability were also studied. Patient’s data was analyzed using the Mann Whitney *U* test, whereas Student’s *t* test was performed to analyze the in-vitro data.

**Results:**

PBMCs from COPD subjects showed a significant decrease in extracellular acidification rate (ECAR) while utilizing glucose as compared to HNS (151.9 Vs 215%). Mitochondrial oxygen consumption rate (OCR) on palmitate or pyruvate was also found to be significantly lower in COPD subjects as compared to HS and a strong positive correlation between palmitate OCR in PBMCs and FEV_1_ (r = 0.74, *p* < 0.05) and FVC (r = 0.79, *p* < 0.05) values in HS was observed. The metabolic shift towards fatty acid metabolism in healthy smokers promoted an inflammatory cytokine response with a greater increase in the levels of IL-5, IL-9 and IFN-α as compared to IFN-γ, IL-17 and TNF-α. In-vitro experiments with RAW 264.7 cells showed similar metabolic alterations and a reduced ability to phagocytose *Streptococcus pneumonia* and *Haemophilus influenza* after cigarette smoke exposure in the presence of glucose or palmitate.

**Conclusions:**

These findings indicate a metabolic basis for the inflammatory response in COPD and could suggest a new therapeutic target for controlling the immune response and delaying the onset of disease.

**Trial registration:**

This observational study was retrospectively registered in the Clinical Trails Registry – India (ICMR – NIMS) on 19th January 2018 with the registration number CTRI/2018/01/011441.

**Electronic supplementary material:**

The online version of this article (10.1186/s12931-019-1139-2) contains supplementary material, which is available to authorized users.

## Introduction

COPD is a chronic, progressive disease of the airways and lung parenchyma due to prolonged exposure to noxious particles, including tobacco smoke and is currently estimated to cause 3.2 million deaths every year [1]. COPD is characterized by repeated exacerbations due to recurrent respiratory tract infections and systemic inflammation, thought to be due to overspill of inflammatory mediators from the lungs and/or the entry of noxious particles into the systemic circulation [2]. While the clinical features of systemic inflammation are well recognized, very little is known about the underlying molecular mechanisms that drive it.

**Fig. 1:**
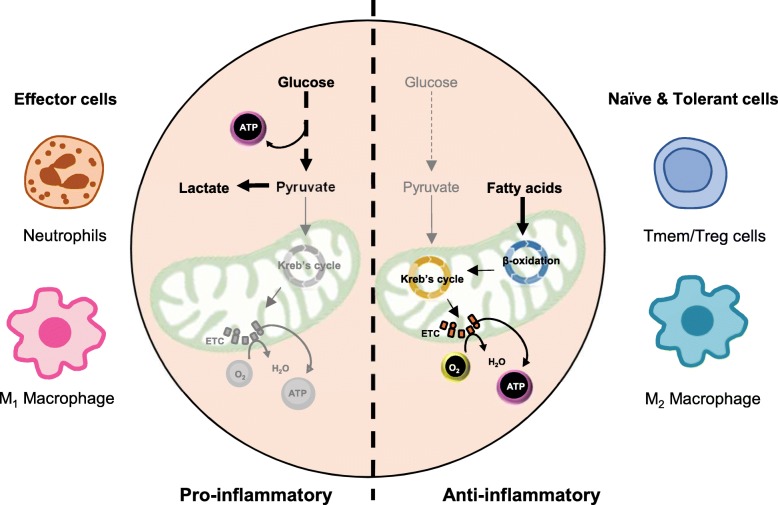
Metabolic changes in immune cells based on inflammation status. Glucose is the principle substrate used by effector cells such as neutrophils and M1 macrophages for the generation of energy. These are also the cells which are the first-responders to initiate an inflammatory response and thus pro-inflammatory in nature. Fatty acid oxidation is the predominant energy generation pathway used by the naïve and tolerant cells such as M2 macrophages and T memory/regulatory cells. These cells are generally involved in resolving the inflammation and thus are anti-inflammatory in nature

Peripheral blood mononuclear cells (PBMCs) comprising of monocytes, lymphocytes and natural killer cells are an important circulating cell population which act as sensors and effectors of metabolic and inflammatory stresses. Glucose and fatty acids are two of the most important substrates utilized by these cells for energy production through glycolysis and β-oxidation of fatty acids. The acetyl-Co A formed through these mechanisms then enters the Krebs cycle where electrons and co-factors are released which are sequentially transferred from one complex to another through the electron transport chain (ETC) leading to oxidative phosphorylation at Complex V. Energy generation in the absence of oxygen or impairment in the Krebs cycle or ETC in the mitochondria happens through the glycolytic pathway and is limited by the amount of ATP produced. The pyruvate formed in this scenario is converted to lactate and extruded out of the cells which leads to acidification of the extracellular milieu (Fig. 1). PBMCs are known to maintain distinct metabolic profiles during various stages of inflammatory stress [3, 4]. They respond to environmental cues by orchestrating their metabolic pathways and laying the groundwork necessary to mount [5–7], maintain [8] or resolve inflammatory responses [9]. Naïve and tolerant cells are known to depend on fatty acid oxidation for the generation of energy [9–11], whereas the effector cells are mainly dependent on aerobic glycolysis for the immediate demand of ATP [6, 12] (Fig. 1).

Our previous animal studies have shown that tobacco smoke [13, 14] and its constituents such as acrolein [15] significantly hamper the ability of mouse alveolar type II cells to metabolize glucose for the generation of energy. This energy deficit seems to be compensated by increasing the fatty acid metabolism through a genetic up-regulation of β-oxidation-related genes (*Acadl, Acadm, Cpt1a* and *Slc25a20*, 13) and supported by the supply of surfactant fatty acids [14] present in the type II alveolar cells. Mitochondria play a central role in this shift because of the localization of all β-oxidation enzymes in the mitochondrial matrix. These animal studies were performed in mice exposed to cigarette smoke for eight weeks which could not be clinically equated to COPD from an inflammation perspective due to lack of neutrophilic infiltration [13]. The metabolic changes observed in the animal studies could at best be expected in healthy smokers. In COPD, the mitochondrial structure has been found to be altered with fragmentation, branching, and loss of cristae, at least in the bronchial epithelial cells [16]. Airway smooth muscle cells in COPD subjects also showed a loss of mitochondrial membrane potential and decrease in oxygen consumption [17] indicating that mitochondrial dysfunction and deregulated metabolic adaptation may be an important component in COPD pathogenesis [18]. However, mitochondrial dysfunction as a systemic consequence of tobacco smoke exposure in COPD subjects has not been studied before. We hypothesized that long-term exposure to tobacco-smoke would alter the metabolic state of PBMCs and interfere with the immune cell function.

### Experimental procedures

#### Participants

Sixteen healthy non-smokers (HNS), 13 healthy smokers (HS – smokers with normal lung function) and 14 tobacco smoke related COPD patients were recruited at the Chest Research Foundation (Pune, India) along with 11 screen- failures (4 – High fasting blood sugar levels, 2 – probable asthmatics, 2 – hypertension, 2 – could not perform spirometry and 1 – exposure history could not be verified). The screen failure subjects were excluded from any further participation or analysis of their blood samples. COPD was diagnosed on the basis of the history of tobacco smoking (at least ten pack-years) and post-bronchodilator spirometry with FEV_1_/FVC ratio < 0.7 as per the GOLD guidelines. All the participants were above the age of 40 years, and those suffering from other comorbid conditions such as diabetes and hypertension were excluded. Also, participants having a history of any other lung condition such as tuberculosis, asthma, interstitial lung diseases were excluded. All COPD subjects were stable when recruited into the study and free from exacerbations for at least three months. 10 ml of blood was collected through venipuncture, of which one ml was used for complete blood count analysis. Patients were in a fasting state and the smokers had abstained from smoking for at least 12 h at the time of blood collection. Plasma samples were stored for inflammatory cytokine analysis. The subjects were informed about their rights associated with their participation in the study along with the risks associated with the procedures. A written informed consent was obtained from all the study participants, and the study was reviewed and approved by the Institutional Ethics Committee of the Chest Research Foundation.

#### Isolation and culture of PBMCs

PBMCs were isolated from 9 ml blood samples collected from study participants using the Percoll-density gradient centrifugation method [19]. Briefly, whole blood was added to 5 ml of 6% Dextran and then diluted with 10 ml 1X Hank’s Balanced Salt Solution (HBSS). This mixture was allowed to settle in order to sediment the red blood cells. While the blood was sedimenting, gradients were prepared using 55, 67 and 81% Percoll solution. The supernatant at the top of sedimented blood, containing PBMCs were then transferred to another tube and centrifuged at 350 g for 10 min. The pellet was resuspended in 55% Percoll solution and layered above the 81 and 67% gradient. This gradient solution was then centrifuged again at 350 g for 30 min with the PBMCs gathering between the top layer of 55% Percoll and the middle layer of 67% Percoll. PBMCs forming a ring between the two layers were then resuspended in 1X HBSS and centrifuged again at 350 g for 10 min. The resulting cell pellet was resuspended in RPMI1640 supplemented with 5.5 mM glucose, 10% FBS and then plated in appropriate culture-plates for further assays. The media was not supplemented with any additional nutrients for overnight culture at 37 °C in 5% CO2 incubator. The cells were confirmed to be PBMCs by staining with Diff-Quik stain. All the chemicals required for isolation and culture of PBMCs were procured from Sigma-Aldrich, St. Louis, MO unless specified otherwise.

#### Extracellular flux analysis

Freshly isolated PBMCs were plated at a density of 150,000 cells/well in the Seahorse XFp cell culture mini plates and allowed to settle overnight. Glucose and pyruvate metabolism was measured in Seahorse base medium, whereas fatty acid metabolism was measured in Krebs-Heinsleit buffer. On the morning of the extracellular flux assay, the culture medium was replaced with Seahorse basal medium without glucose or pyruvate and the cells were starved for ~40mins till the Xfp plates are calibrated and the cells are incubated in a non-CO_2_ environment as per the standard Seahorse protocol. The assay protocol was modified to measure initial basal respiration (3 readings) and then glucose (10 mM), Palmitate-BSA (200 μM) or pyruvate (2 mM) was added through the first port and the change in mitochondrial oxygen consumption rate (OCR) and extracellular acidification rate (ECAR) was measured using the Agilent Seahorse XFp Extracellular Flux Analyzer (Agilent Technologies, USA) as described before [14, 15]. Four micrometer oligomycin was added through the second port to measure the ATP linked respiration. One micrometer FCCP, added through the third port uncoupled the mitochondria and the maximal and spare respiratory capacity were measured. Specific inhibitors in the form of 2-deoxyglucose (50 mM), etomoxir (440 μM) or rotenone (1 μM) was added through the fourth port to inhibit complete mitochondrial respiration on glucose, palmitate or pyruvate, respectively. OCR represents the consumption of oxygen at Complex IV of the ETC while metabolizing glucose/palmitate-BSA/pyruvate. ECAR represents the acidification of the extracellular environment due to formation of lactate (while metabolizing glucose) or carbon dioxide (through the Krebs cycle). The cells were washed 2X after the OCR and ECAR measurements with 1X PBS pre-warmed to 37 °C before measuring the protein concentration using the Bradford Assay to normalize the values.

Palmitate was conjugated with fatty acid-free bovine serum albumin (BSA) by dissolving in 150 mM NaCl solution at 70 °C with stirring. The conjugation was performed at 37 °C in 5 ml aliquots with stirring and unconjugated BSA solution was also used as a control for all the measurements to confirm if the change in OCR was due to palmitate.

RAW264.7 cells were seeded at a density of 40,000 cells per well and the analysis was performed by exposing the cells to CSC in the Seahorse Xfp plates directly, in the presence of complete medium containing glucose and pyruvate. After the CSC exposure for 1 h, the medium was changed to Seahorse basal medium (without glucose and pyruvate) and the plate was incubated in the non-CO_2_ incubator for ~ 40 mins. In order to analyze the response of RAW 264.7 cells to glucose or fatty acids, the substrates were added directly from the first port and the change in ECAR and OCR was measured in real-time, respectively. All values were normalized to the protein concentration in the well after the flux analysis.

Data is represented as percent change in OCR and ECAR in order to compare the response to glucose or fatty acids from multiple subjects and across multiple groups. This percentage change was determined by considering the value just before the addition of substrate (Reading number 3, Additional file 1: Figure S1A, B) as Pre and the highest value reached while after the addition of the substrate (Post). The % change was determined using the following formula:

$$ \%\mathrm{change}=\left[\left(\mathrm{Post}-\mathrm{Pre}\right)/\mathrm{Pre}\right]\ \mathrm{X}\ 100 $$

ATP-linked respiration was measured as the difference between OCR on oligomycin and OCR on glucose (Additional file 1: Figure S1A) or palmitate. The maximal respiratory capacity was the highest % change in OCR realized after addition of FCCP and the difference between the maximal respiratory capacity and OCR on glucose (Additional file 1: Figure S1A) or palmitate represented the spare respiratory capacity.

Glycolytic capacity was measured as the maximum ECAR reached after inhibition of mitochondrial respiration (oligomycin) and the difference between the maximum ECAR and the ECAR after addition of glucose was represented as glycolytic reserve capacity. ECAR after addition of 2-DG was considered as the non-glycolytic ECAR (Additional file 1: Figure S1B).

#### Cytokine analysis

Plasma samples were collected and stored from one ml of peripheral blood at − 80 °C. For cytokine analysis, plasma samples were thawed and centrifuged at 10,000 X g for 10 min at 4 °C. The samples were then transferred to a new tube and diluted 8 times for further analysis by flow cytometry using kits available from Mitenyi Biotech, CA, USA as per manufacturers protocol. The assay utilizes capture beads coated with antibodies to specific cytokines. Fluorescence-conjugated secondary antibodies were then utilized against specific cytokines to quantify them using a flow cytometer.

#### Cell-culture and cigarette smoke exposure

RAW264.7 cells were obtained from American Type Cell Culture and cultured in Dulbecco’s Modified Eagle Medium (DMEM) supplemented with 10% FBS. Cigarette smoke condensate (CSC) was prepared by pumping cigarette smoke from 4 commercially available cigarettes (Kings Gold Flake, ITC Ltd.) into a condensation apparatus. The resultant condensate was then filtered using 0.2 μm membrane filter (Pall Life Sciences, Port Washington, NY). The condensate was standardized spectrophotometrically (Multiskan spectrum, Thermo Scientific, Waltham, MA) by measuring the UV absorbance at 400 nm. Cells were exposed to CSC for 1 h after which they were washed or scrapped based on further experiments.

#### Cell viability assay

Cell viability was measured using 3-(4,5-Dimethylthiazol-2-yl)-2,5-diphenyltetrazolium bromide (MTT) as described previously [15]. RAW264.7 cells were seeded at a density of 80,000 cells per well in a 96-well plate and allowed to settle overnight. Cells were exposed to CSC in media containing glucose (10 mM) and pyruvate (2 mM), glucose (10 mM) or palmitate-BSA (200 μM) only.

#### Mitochondrial membrane potential and ROS assays

Mitochondrial membrane potential was measured using 5,5′,6,6′-tetrachloro-1,1′,3,3′-tetraethylbenzimidazolyl-carbocyanine iodide (JC-1; Invitrogen, Inc., Waltham, MA). Briefly, RAW264.7 cells were incubated with 10 μg/ml of JC-1 dissolved in sterile warmed PBS after exposure to CSC for 1 h either in the presence of complete medium or palmitate. The cells were incubated for 10 min after which they were washed and imaged using a fluorescence microscope (Motic, Hong Kong). JC-1 is a cationic dye which is known to accumulate in the mitochondrial by forming red aggregates whereas the monomeric (green) form remains in the cytoplasm. The ratio of red to green fluorescence was used to indicate the mitochondrial membrane potential. The images were analyzed using ImageJ.

Mitochondrial ROS was measured using MitoSOX Red (Invitrogen, Inc., Waltham, MA). Cells were exposed to CSC either in complete medium or palmitate and then washed with warm sterile PBS. Five micrometer MitoSOX was added to the wells and incubated for 10 min at 37 °C. The wells were then imaged using the red filter on a fluorescence microscope (Motic, Hong Kong). The images were analyzed using ImageJ.

#### Phagocytosis assays

The ability of RAW264.7 cells to phagocytose heat-killed *Streptococcus pneumonia* and *Haemophilus influenza* was measured as described previously [20]. Briefly, cells were seeded at a density of 80,000 cells per well overnight and exposed to CSC the next day in complete medium for 1 h. Bacteria were labelled with Alexa Fluor 488 and incubated with cells in the presence of complete medium containing glucose (10 mM) and pyruvate (2 mM)), or glucose (10 mM) or fatty acids (Palmitate-BSA, 200 μM) only for 3 h to measure their uptake in the presence of different substrates. The bacteria which was not phagocytosed was removed by washing the wells with warm PBS. The fluorescence emitted from bacteria attached to the cell surface was quenched using trypan blue (1% w/v). The wells were then imaged for internalized bacteria using a fluorescence microscope (Motic, Hong Kong) and analyzed using ImageJ. Cell viability was also measured after the phagocytosis assay to confirm that the change in phagocytosis was not due to changes in cell number.

#### Glyceraldehyde-3-phosphate dehydrogenase (GAPDH) enzyme activity

GAPDH enzyme activity was measured in PBMCs and RAW264.7 cells lysed in RIPA buffer supplemented with protease inhibitor as described previously [13, 15]. Briefly, the formation of NADH in the presence of the enzyme (samples) and substrates was monitored using a UV-spectrophotometer at 340 nm.

#### Statistical analyses

The demographic data is summarized in Table 1. Normality test (one sample Kolmogorov Smirnov test) was performed for all the parameters before applying any statistical tests. Most of the patient data was non-normally distributed and thus non-parametric tests were used for all the data analysis. One-way ANOVA with post hoc analysis was used to compare mean differences between different study groups, namely HNS, HS and COPD for all the data in Table 1. For non-normally distributed, the non-parametric Kruskal-Wallis test was applied for comparing differences between different study groups. Post-hoc test for non-normally distributed data was performed using Mann-Whitney test. Data is represented by median ± interquartile range values in the figures and the results section. Correlation between spirometry and metabolic parameters were calculated using Pearson correlation coefficient. Statistical significance was reported at *p* < 0.05 level. All the statistical computation was performed using Statistical Package for Social Sciences (SPSS) Version 22.0.

**Table 1:** Demographic details and baseline characteristics of study participants

	HNS (*n* = 16)	HS (*n* = 13)	TS-COPD (*n* = 14)	*p*-value
Age (y)	53.38 ± 10.43	60.15 ± 8.754	68.36 ± 8.924	< 0.001
Gender (M/F)	8 M/8F	13 M/0F	14 M/0F	N/A
BMI	24.31 ± 3.909	23.71 ± 2.814	19.75 ± 3.12	< 0.01
Fasting BSL (mg/dL)	88.63 ± 12.1	91.54 ± 16.22	95.31 ± 11.68	NS
Smoking history, Pack years (Ex:Current)	N/A	32.69 ± 17.37 (2:11)	39.45 ± 29.98 (6:8)	NS
FEV_1_ (% Predicted)	104.3 ± 10.57	97.72 ± 9.394	51.09 ± 26.24	< 0.001
FVC (% Predicted)	102.3 ± 13.3	95.08 ± 11.46	85.85 ± 20.78	< 0.05
FVC/FEV_1_%	102.5 ± 6.387	103.3 ± 4.426	58.12 ± 19.49	< 0.001
COPD gold stage (I/II/III/IV)	N/A	N/A	3/2/6/3	N/A
Monocytes (%)	5.786 ± 0.699	7.5 ± 1.446	7.556 ± 1.667	< 0.01
(/μL)	435.9 ± 119	595.6 ± 148.9	619.4 ± 224	< 0.05
Neutrophils (%)	55.93 ± 12.39	53.58 ± 8.051	60.44 ± 6.966	NS
(/μL)	4186 ± 1170	4390 ± 1431	5033 ± 1656	NS
Lymphocytes (%)	34.43 ± 10.41	34.83 ± 7.158	29.11 ± 5.667	NS
(/μL)	2612 ± 1124	2810 ± 792.1	2359 ± 705.4	NS
Eosinophils (%)	3.857 ± 2.685	4.083 ± 3.37	2.889 ± 1.833	NS
(/μL)	280.3 ± 194.4	345.8 ± 371.1	266.2 ± 260.8	NS

Data is represented as mean ± standard deviation. *HNS* Healthy non-smokers, *HS* Healthy smokers and *TS-COPD* Tobacco-smoking – COPD subjects. ANOVA statistical analysis was performed and *p* < 0.05 was considered significant. *NS* Non-significant and *N/A* Not applicable

For normally distributed data, Student‘s *t* test assuming unequal variances was performed as indicated in the figure legends. Data has been represented by mean ± standard deviation from a minimum of three experiments.

## Results

The demographic details of 16 healthy non-smokers, 13 healthy smokers and 14 COPD subjects participating in the study are indicated in Table 1. As expected, COPD subjects were older, with a lower BMI and a significantly lower lung function as compared to healthy subjects. The smoking history was well-matched between healthy smokers and COPD subjects. Complete blood count analysis indicated no significant changes in the absolute and percentage count of eosinophils, neutrophils, and lymphocytes between all the three groups. The absolute count (percentage) of monocytes in healthy non-smokers was 435.9 ± 119 cells/μL (5.7 ± 0.6%) which was significantly lower than 595.6 ± 148.9 cells/μL (7.5 ± 1.4%) and 619.4 ± 224 cells/μL (7.5 ± 1.6%) among healthy smokers and smoker COPD subjects, respectively (Table 1).

### Impaired glucose metabolism in COPD subjects

**Fig. 2:**
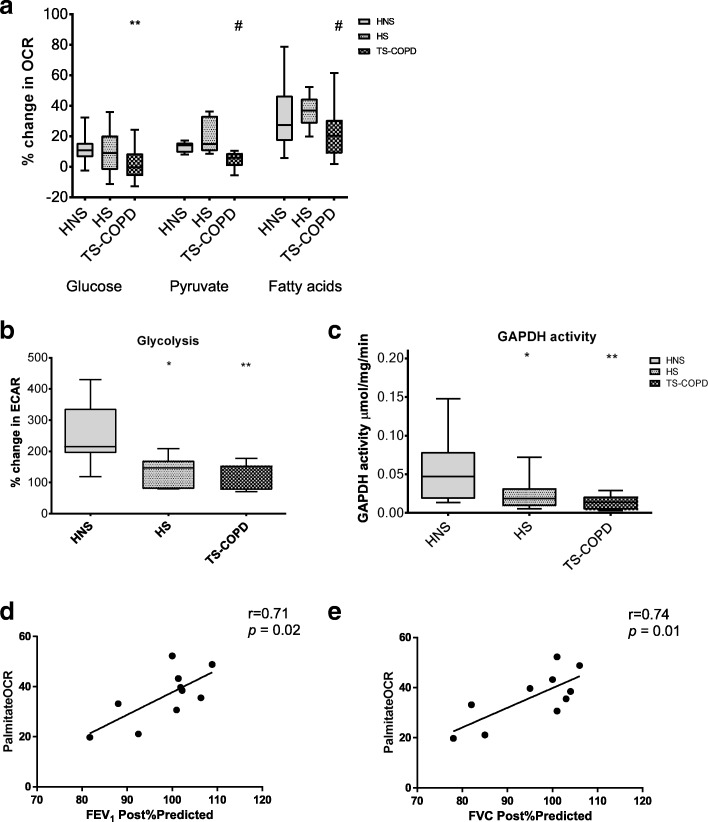
Defective glycolytic and mitochondrial metabolism in PBMCs of COPD subjects. % Change in OCR (**a**) in PBMCs from 16 healthy non-smokers (HNS), 10 healthy smokers (HS) and 13 tobacco smoking COPD (TS-COPD) subjects while metabolizing glucose (10 mM), pyruvate (2 mM) and palmitate-BSA (200 μM) measured using the XF Extracellular Flux analyzer as described in the material and methods section. % Change in ECAR (**b**) in PBMCs while metabolizing glucose (10 mM) was measured as described in the material and methods section. **c** GAPDH enzyme activity measured by monitoring consumption of NAD at 340 nm in 15 HNS, 13 HS and 10 TS-COPD subjects. Median values are indicated with a line for each group and * *p* < 0.05, ** *p* < 0.01 vs HNS; # *p* < 0.05 Vs HS was considered as statistically significant using the Mann-Whitney U test. Correlation analysis of fatty acid metabolism with lung function parameters showed a strong positive correlation between palmitate OCR and FEV_1_ (D) and FVC (E) for 10 healthy smokers as shown

The ability of PBMCs to metabolize glucose was studied by measuring the % change in OCR and ECAR from baseline (without glucose). PBMCs from COPD subjects (− 0.24 ± 14.6%) showed a significantly lower OCR as compared to healthy non-smokers (10.8 ± 9.3; *p* < 0.05) (Fig. 2a). ATP production and spare respiratory capacity were found to decrease by 17% (*p* < 0.5) and 34% (*p* < 0.01) respectively in HS as compared to HNS (Table 2). PBMCs from COPD patients showed a significant decrease in ATP production, spare and max respiratory capacity by 10% (*p* < 0.01), 21% (*p* < 0.05) and 22% (*p* < 0.05), respectively as compared to HNS (Table 2) while metabolizing glucose.

**Table 2:** Effect of electron transport chain complex inhibitors on OCR and ECAR in PBMCs

		HNS	HS	TS-COPD
Glucose OCR	ATP Production (%)	61.3 (56.5–66.2)	50.7 (48.6–59.6)^*^	54.8 (48.8–57.3)^**^
	Max Resp. Capacity (%)	91.9 (73.8–109.5)	80.2 (51.6–93.8)	72.1 (59.0–75.7)^*^
	Spare Resp. Capacity (%)	87.5 (61.6–105.4)	57.1 (42.7–69.0)^**^	68.0 (57.8–77.3)^*^
Glucose ECAR	Glycolytic Capacity (%)	315.6 (278.7–358.1)	194 (170–267.8)	198.2 (144.4–219.1) ^*^
	Glycolytic Reserve (%)	92.4 (65.8–102.6)	84.3 (72.2–110.6)	57.8 (49.7–64.5)^*,##^
	Non-Glycolytic Acidification (%)	73 (63.4–80.3)	74.5 (65.7–92)	94.4 (83.9–99.9)^**^
Palmitate OCR	ATP Production (%)	53.5 (47.9–72.4)	62.5 (52.7–75.9)	37.7 (31.4–45.8)^***, ##^
	Max Resp. Capacity (%)	101.6 (94.3–118.9)	104.2 (98.3–130.7)	83.1 (56.3–86.5)^**, ##^
	Spare Resp. Capacity (%)	66.7 (47.6–81.1)	65.3 (61.5–95.6)	38.5 (32.4–57.1)^*, #^
	Non-fatty acid Mitochondrial Resp. (%)	30.8 (19.5–42.3)	38.6 (30.6–49.5)	37.4 (25.6–54.8)

Data for ATP production, maximum respiratory capacity, spare respiratory capacity, glycolytic capacity and glycolytic reserve is represented as median % change in OCR/ECAR (Inter-quartile range). Non-glycolytic acidification and non-mitochondrial respiration is represented as percentage of baseline (before adding glucose or palmitate) ECAR and OCR, respectively. *HNS* Healthy non-smokers, *HS* Healthy smokers and *TS-COPD* Tobacco-smoking COPD subjects. * indicates *p* < 0.05, ** indicates *p* < 0.01 and *** indicates *p* < 0.001 as compared to HNS whereas, # indicates *p* < 0.05 and ## indicates *p* < 0.01 as compared to HS using the Mann-Whitney U test for between-group comparison

The significantly lower levels of ECAR in HS (146.8 ± 141.9%, *p* < 0.5) and COPD (151.9 ± 77.2%, *p* < 0.01) patients as compared to HNS (215 ± 141.9%) also indicated a reduced ability to utilize glucose (Fig. 2b). This was further confirmed with a reduced enzyme activity of the glycolytic enzyme; Glyceraldehyde-3-Phosphate Dehydrogenase (GAPDH) in HS (0.018 ± 0.022 μmol/min/mg of protein; *p* < 0.05) and COPD subjects (0.013 ± 0.016 μmol/min/mg of protein; *p* < 0.01) as compared to HNS (0.046 ± 0.06 nmol/min/mg of protein). COPD patients also showed a significant decrease in maximum glycolytic capacity (37%, *p* < 0.05) and the glycolytic reserve (37%, *p* < 0.05) and a 29% increase in non-glycolytic acidification (*p* < 0.01) as compared to healthy non-smokers (Table 2).

### Impaired pyruvate metabolism in COPD subjects

The metabolism of pyruvate which is a direct substrate for the mitochondria, was found to be significantly lower in PBMCs of COPD subjects (5.9 ± 8.5%; *p* < 0.05) as compared with healthy smokers (14.9 ± 23%, Fig. 2a).

### Impaired fatty acid metabolism in COPD subjects

COPD subjects showed a significantly reduced ability to metabolize fatty acids as measured by the % change in baseline (without Palmitate-BSA) oxygen consumption rate (OCR) after addition of palmitate-BSA as compared to healthy smokers (20.3 ± 22.1% Vs 36.9 ± 16.3%; *p* < 0.05) (Fig. 2a). The higher rate of fatty acid metabolism in healthy smokers correlated significantly and positively with lung function parameters; FEV_1_ (r = 0.74, *p* < 0.05) (Fig. 2d) and FVC (r = 0.79, *p* < 0.05) (Fig. 2e). COPD patients showed a significant decrease in ATP production, spare and max respiratory capacity by 29% (*p* < 0.001), 18% (*p* < 0.01) and 42% (*p* < 0.05), respectively as compared to HNS (Table 2) and 39% (*p* < 0.01), 20% (*p* < 0.01) and 41% (*p* < 0.05), respectively as compared to HS while metabolizing palmitate (Table 2).

### Inflammatory cytokine response in COPD patients

**Fig. 3:**
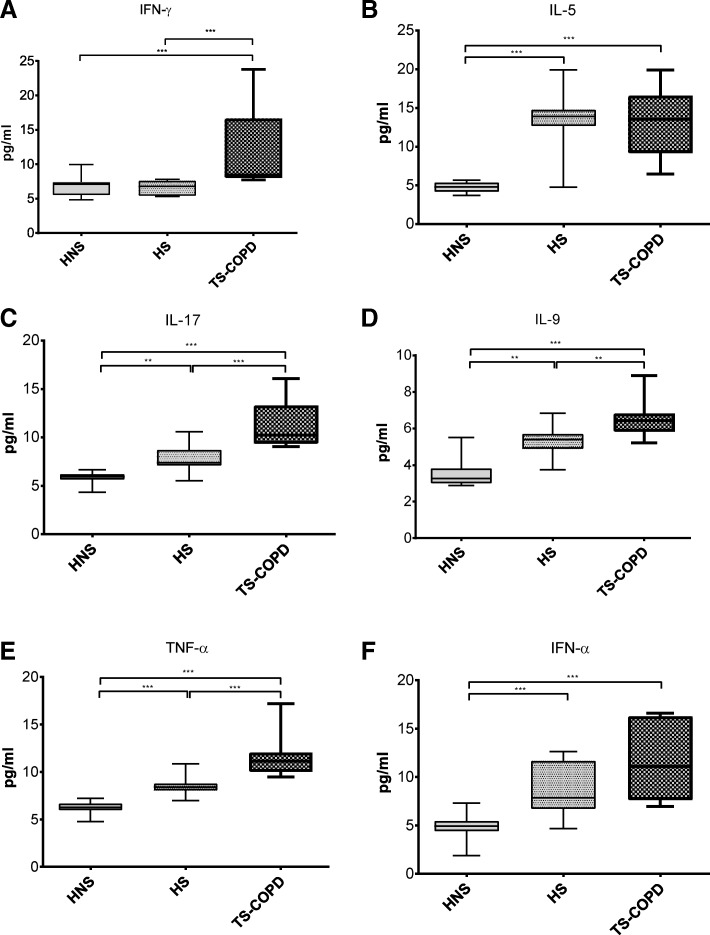
Changes in plasma inflammatory cytokine levels in healthy smokers and COPD subjects*.* Plasma cytokine levels of IFN-γ (**a**), IL-5 (**b**), IL-17 (**c**), IL-9 (**d**), TNF-α (**e**) and IFN-α (**f**) were measured as described in the material and methods section in 15 HNS, 12 HS and nine TS-COPD subjects. ** *p* < 0.01 and *** *p* < 0.001 as compared using the Mann-Whitney *U* test

Plasma levels of inflammatory cytokines (IFN-γ, IL-17, TNF-α, IL-5, IL-9 and IFN-α) were measured in the subject population using flow cytometry. The levels of all the inflammatory cytokines; IFN-γ, IL-17, TNF-α, IL-5, IL-9 and IFN-α were significantly elevated in COPD subjects as compared to HNS (*p* < 0.001) (Fig. 3a-f). HS subjects also showed a significant increase in all the cytokines as compared to HNS, except IFN-γ (Fig. 3a-f).

In COPD subjects, the median fold change in IL-5 – 2.82, IL-9 – 1.96 and IFN-α – 2.25 was greater than IFN-γ – 1.19, IL-17 – 1.73 and TNF-α – 1.77 as compared to HNS. Similarly, the median fold change in IL-5 – 2.89, IL-9 – 1.64 and IFN-α – 1.59 was greater than IFN-γ – 0.96, IL-17 – 1.25 and TNF-α – 1.34 in HS as compared to HNS.

### Cigarette smoke induced cellular and mitochondrial toxicity

**Fig. 4:**
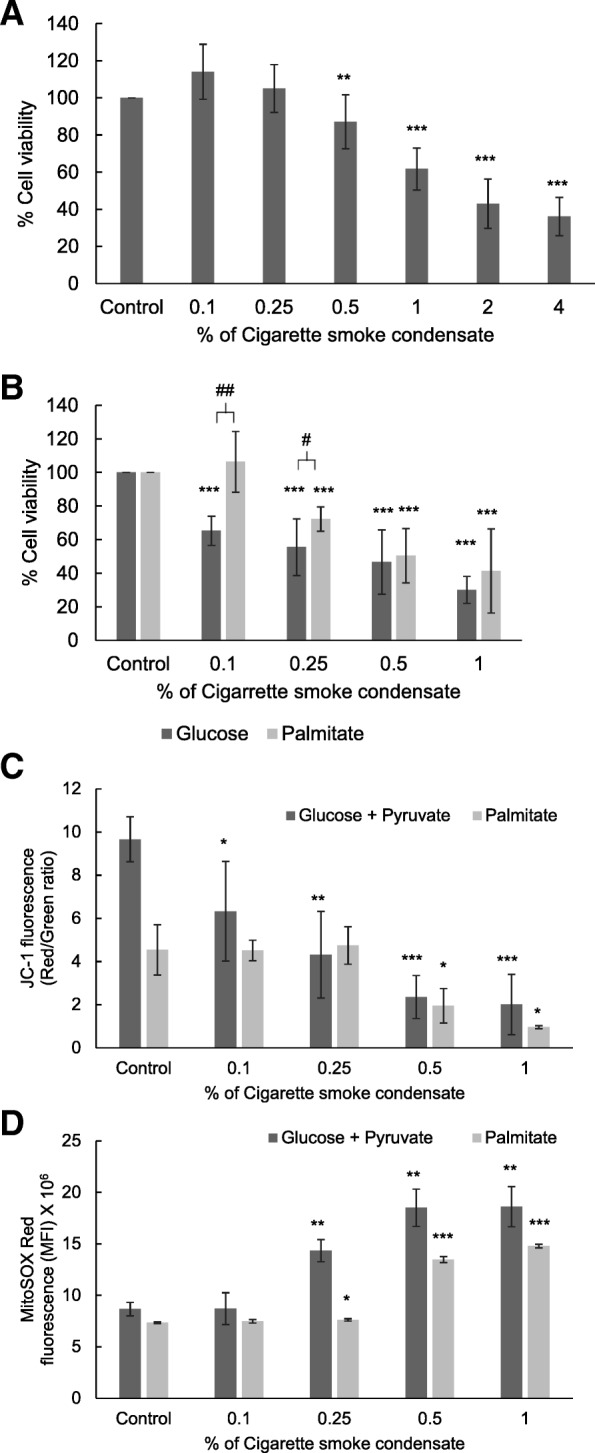
CSC induced cellular and mitochondrial toxicity in mouse macrophage cells. The cytotoxicity of CSC was studied by measuring the ability of the cells to reduce 3-(4,5-dimethylthiazol-2-yl)-2,5-diphenyl tetrazolium bromide (MTT) as described in the material and methods section in the presence of complete medium (glucose and pyruvate, **a**) or glucose and palmitate-BSA individually (**b**). Mitochondrial membrane potential was measured using the JC-1 dye after CSC exposure as described in the materials and methods section in the presence of complete medium or palmitate and is expressed as the ratio of red to green fluorescence (**c**). Mitochondrial superoxide production was measured using the MitoSOX Red dye after CSC exposure as described in the materials and methods section in the presence of complete medium or palmitate and is expressed as the change in mean fluorescence intensity (MFI) (**d**). * *p* < 0.05, ** *p* < 0.01 and *** *p* < 0.001 as compared to their respective control using the *Student’s t*-test. # *p* < 0.05, and ## *p* < 0.01 as compared using the *Student’s t*-test

The effect of 1 h CSC exposure on cell viability was studied by measuring the ability of cells to reduce MTT. A dose-dependent decrease was found in cell viability (Fig. 4a) when supplemented with complete medium containing glucose and pyruvate. Cell viability was also found to decrease significantly and in a dose-dependent fashion after CSC exposure, when supplemented with glucose alone (Fig. 4b). On the other hand, palmitate supplementation protected the cells against CSC toxicity at lower concentrations of 0.1 and 0.25% (Fig. 4b).

Mitochondrial health was studied by measuring the changes in mitochondrial membrane potential and the formation of mitochondrial superoxide after CSC exposure. A dose-dependent mitochondrial membrane depolarization was observed after 1 h CSC exposure with a 79% decrease in the ratio of red to green fluorescence after 1% CSC exposure when supplemented with glucose and pyruvate (Fig. 4c). Palmitate supplementation did not alter the mitochondrial membrane polarization in RAW 264.7 cells after exposure to 0.1 and 0.25% CSC. However, a 78% decrease in the ratio of red to green fluorescence was observed after 1% CSC exposure. Mitochondrial ROS production was also found to be elevated in a dose-dependent manner with increasing concentration of CSC exposure (Fig. 4d). A 114% increase in fluorescence intensity for MitoSOX Red was observed in RAW 264.7 cells after exposure to 1% CSC (Fig. 4d) when supplemented with complete media and 101% increase was observed when supplemented with palmitate (Fig. 4d).

### Cigarette smoke induced alterations in cellular metabolism

**Fig. 5:**
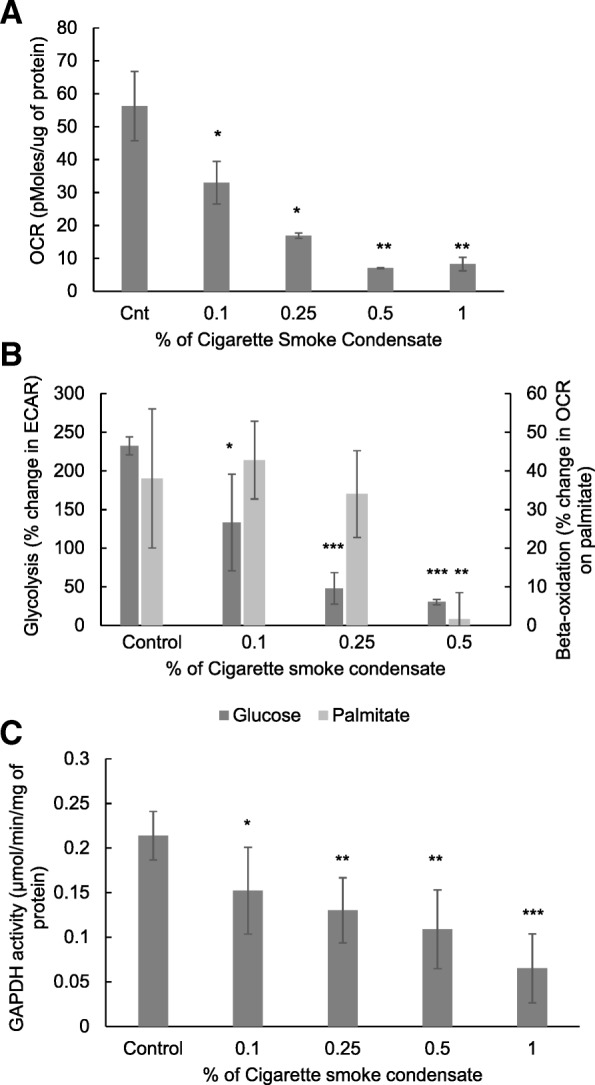
CS exposure alters cellular metabolism in mouse macrophage cells. % Change in OCR (**a**) in RAW 264.7 cells after exposure to CSC for 1 h in the presence of glucose (10 mM) and puyruvate (2 mM) was measured using the XF Extracellular Flux analyzer as described in the material and methods section. % Change in OCR (Palmitate-BSA, 200 μM) and ECAR (glucose 10 mM)) in RAW 264.7 cells after exposure to CSC for 1 h (**b**). GAPDH enzyme activity was measured by monitoring consumption of NAD at 340 nm in RAW 264.7 cellular lysates after exposure to CSC for 1 h as described in the materials and methods section (**c**). * *p* < 0.05, ** *p* < 0.01 and *** *p* < 0.001 as compared to their respective control using the *Student’s t*-test

Mitochondrial respiration (OCR) was found to decrease significantly in a dose-dependent manner with CSC exposure in the presence of complete medium containing glucose and pyruvate (Fig. 5a). 0.1 and 0.25% CSC exposure decreased the ECAR while metabolizing glucose by 42.6 and 79.3%, whereas the OCR while metabolizing palmitate did not change significantly (Fig. 5b). 0.5% CSC exposure decreased glucose ECAR and palmitate OCR by 87 and 95.8%, respectively (Fig. 5b).

A dose-dependent decrease in GAPDH enzyme activity was observed after 1 h CSC exposure. 1% CSC exposure led to a 71.4% decrease in GAPDH enzyme activity (Fig. 5c) further confirming the CS-induced inhibition of glucose metabolism.

### Cigarette smoke induced alterations in macrophage phagocytosis

**Fig. 6:**
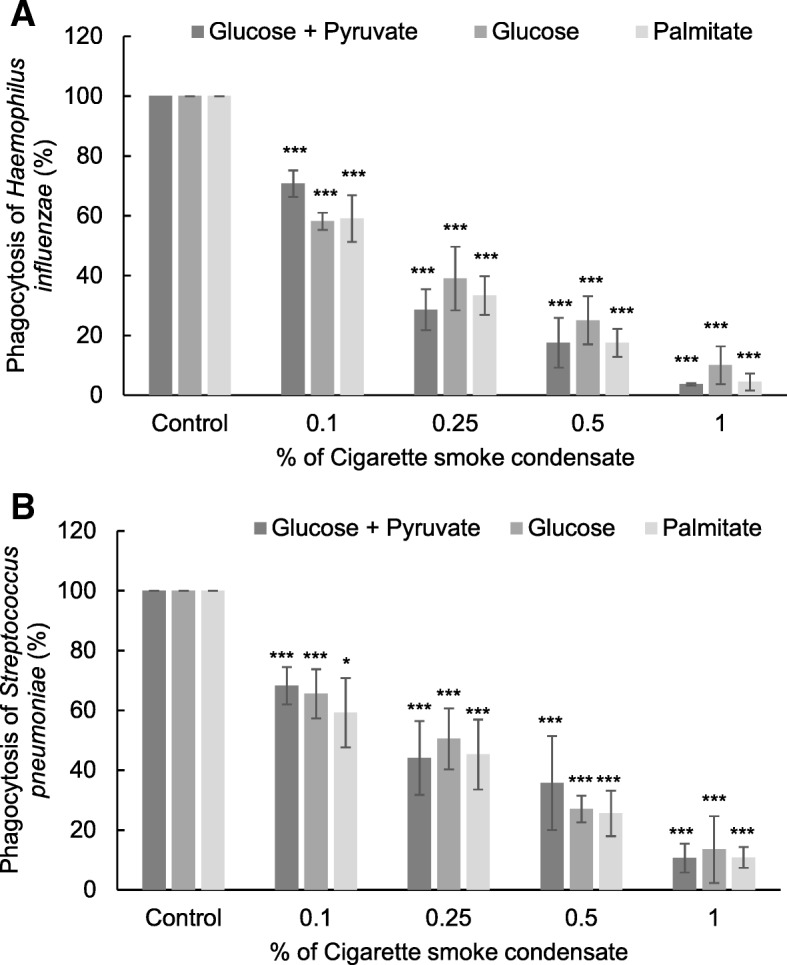
CS exposure impairs bacterial phagocytosis in mouse macrophage cells. % Change in internalization of fluorescently labelled *H. influenza* (**a**) and *S. pneumonia* (**b**) by RAW 264.7 cells after exposure to CSC for 1 h was measured in the presence of glucose (10 mm) and pyruvate (2 Mm), glucose only (10 Mm) or palmitate-BSA (200 μM) using a fluorescence microscope. * *p* < 0.05 and *** *p* < 0.001 as compared to their respective control using the *Student’s t*-test

The effect of CSC exposure on *S. pneumonia* and *H. influenza* phagocytosis by RAW264.7 macrophages was measured using fluorescence microscopy. A dose-dependent decrease in bacterial internalization was observed with 1 h CSC exposure (Fig. 6a, b). Supplementing the cells with different substrates for energy production did not alter the ability of RAW264.7 cells to phagocytose *S. pneumonia* and *H. influenza*.

## Discussion

Immune cells are capable of utilizing both glucose and free fatty acids to generate energy. Pro-inflammatory immune cells generally utilize glucose as the main fuel, whereas anti-inflammatory immune cells predominantly utilize free fatty acids [6, 10, 12] (Fig. 1). The ability to utilize both these fuels is, therefore, necessary to initiate, maintain and resolve an inflammatory response. In this study, we report for the first time that circulating mononuclear cells of subjects with COPD have a reduced ability to utilize glucose, pyruvate or fatty acids at baseline; while circulating mononuclear cells from healthy smokers with preserved lung function have impaired glycolysis, but preserved pyruvate and fatty acid metabolism. The rate of fatty acid metabolism in healthy smokers correlated positively with lung function parameters. These alterations in immune cell metabolism help in their survival but probably interfere with their function and may be responsible for the type of inflammatory cytokine response in the systemic microenvironment in healthy smokers and COPD patients. These results suggest that an impairment in glycolysis and fatty acid oxidation may play an important role in the pathogenesis of COPD and could be explored as a potential target to delay the onset of disease.

Glucose serves as an important source of energy for immune cells because of its fast metabolism and release of energy [12, 21]. PBMCs from COPD subjects and RAW264.7 cells exposed to CSC were found to have an impaired ability to utilize glucose as indicated by the limited substrate supply in the form of pyruvate to the mitochondria (Fig. 2a), lower rates of extracellular acidification through formation of lactate (Fig. 2b, Fig. 5b) and the decrease in activity of the glycolytic enzyme, GAPDH (Fig. 2c, Fig. 5c). GAPDH is a central glycolytic enzyme with a redox-sensitive cysteine in its active site which is susceptible to oxidative stress-induced post-translational modification; S-glutathionylation as observed earlier in mice lung cells exposed to CS [13]. The extracellular acidification mainly occurs due to the protons generated from two sources; glycolysis and tri-carboxylic cycle (TCA, CO_2_ generated is converted to HCO_3_^−^ + H^+^) in the mitochondria [22]. The increase in non-glycolytic acidification in response to addition of 2’deoxy-glucose (Table 2) indicates a probable increase in CO_2_ generated from the TCA cycle. This could be supported by an increase in metabolism of residual cellular amino acids such as glutamine or fatty-acids. However, the reduced OCR on palmitate indicates a defect in the ETC which limits the amount of energy generated through these energy sources. Another minor but possible and relatively unexplored source of CO_2_ is the non-glycolytic breakdown of glucose through the pentose phosphate pathway or the glucoronic acid-xylulose cycle which splits C-1 or C-6 as CO_2_. A strong glycolytic response is usually considered a hallmark for immune cell activation and for phagocytosis in macrophages, and an impairment in glucose metabolism may predispose these cells from HS and COPD patients to an attenuated immune response and reduced phagocytosis.

The lower rate of fatty acid metabolism in COPD subjects (Fig. 3a) and RAW264.7 cells exposed to higher concentrations of CSC could most likely be due to an inability of the PBMC mitochondria to oxidize fatty acids due to mitochondrial damage. This is supported by the decrease in ATP production, maximum and spare respiratory capacity of PBMC mitochondria from COPD subjects while metabolizing palmitate as compared to HNS and HS (Table 2). A reduced activity of the mitochondrial ETC due to cigarette smoke exposure has also been reported earlier in the PBMCs [23] and lymphocytes [24]. Moreover, an overall reduction in oxygen delivery due to impaired lung function would also make β-oxidation a challenging task.

The higher rate of fatty acid metabolism in healthy smokers as compared to COPD subjects (Fig. 2a); as also reported by us earlier in mice exposed to CS [14], correlated positively with lung function parameters (Fig. 2d and e) suggesting that higher fatty acid metabolism may be a protective mechanism that prevents the development of COPD. As the lung function is known to decline with age, healthy smokers with lower FEV_1_ [25] and lower fatty acid metabolism could warrant a close follow-up to see if they are more susceptible to developing COPD in the near-term. Previous studies have identified the usefulness of fat-rich diet and higher BMI as protective factors associated with relatively better lung function [26] and mortality [27] outcomes, respectively. Cells supplemented with fatty-acids showed a better ability to respond to CSC-induced stress as indicated by the higher cell-viability which may be attributed to the shift in ATP generation from glucose to fatty acids. Exposure to higher concentrations of CSC damages the mitochondria which may prevent the further utilization of fatty acids as an energy source (Fig. 4b). We found that the membrane potential is maintained (Fig. 4c) and mitochondrial ROS production (Fig. 4d) is not increased even after exposure to 0.25% of CSC under palmitate supplementation. These data indicate that palmitate supplementation offers an alternative source for energy production and helps to prevent mitochondrial toxicity.

In concordance with our results, Shaykhiev et al. [28] observed a deactivation of the M1-related genes in healthy smokers that primarily depend on glucose along with consequent induction of the M2-related genes that are primarily dependent on fatty acids. Interestingly, CD36, which is involved in the uptake of fatty acids into the cells was one of the M2-related genes which were found to be significantly up-regulated in the healthy smokers [28].

The reduction of OCR and ECAR in COPD PBMCs is most likely because of inhibition of enzymes of the glycolytic and β-oxidation pathway which may lead to an accumulation of the intermediates within the cells. A number of glycolytic enzymes in immune cells are known to perform non-metabolic functions through signaling and gene regulation that influence the inflammatory response [29]. For eg, GAPDH is known to be translocated to the nucleus under conditions of oxidative stress to initiate apoptosis [30] or induce translation of IFN-γ and IL-2 in T-cells under glucose deprived conditions [31]. Alternative pathways in the cytoplasm may also metabolize glucose such as the pentose phosphate pathway (which is also known to be upregulated under oxidative-stress) and glucoronic acid pathway. Unutilized or excess fatty acids may uncouple mitochondrial respiration and induce lipotoxicity if not stored in the form of lipid droplets which act as highly dynamic storage pool of fatty acids to support immune cell activation and function. Similarly, unutilized glucose could also be stored as glycogen within the cells. Thus it could be postulated that, impaired immune cell metabolism may drive disease pathophysiology by altering the immune cell function. As is evident from both, in-vitro and in-vivo data; mitochondrial health in immune cells play an important role in the progression of COPD (Fig. 2a, Fig. 4b) and could be considered as the driving force in the development of COPD. As long as the mitochondria are healthy, the disease progression is impeded as shown in healthy smokers and RAW 264.7 cells exposed to 0.1 and 0.25% CSC.

Immunometabolic reprogramming is known to be at the center of an immune response to an inflammatory stimulus [7, 32]. Glucose metabolism supports the acute response to inflammatory stimuli with the production of instantaneous energy required for immune cells’ migration [33] and activation [21]. The anti-inflammatory/resting state is characterized by a metabolic shift from glucose to fatty acid metabolism in tolerant/naïve cells for energy production [9, 11]. A number of studies have tried to define the macrophage polarization in COPD patients as either M1 or M2 based on gene expression or cytokine profiles with conflicting results [28, 34, 35]. As cellular metabolism is intricately linked to pro- or anti-inflammatory phenotype of immune cells, we aimed to validate the immune cell phenotype with its metabolic state. This was confirmed in healthy smokers, where the lower rates of glycolysis along with preserved fatty acid metabolism promoted a Th2 type immune response [10, 36] as shown by a greater increase in the Th2 cytokines (IL-5 and IL-9) in the plasma (Fig. 3b, d). Th2 cytokines are known to promote the M2 phenotype in macrophages [37, 38] which is primarily dependent on fatty acid metabolism. These findings are consistent with other studies which have shown similarly elevated levels of blood cytokines in COPD patients [39, 40].

Macrophages are an important immune cell population in COPD as they are responsible for initiating and resolving inflammation and maintaining lung homeostasis by clearing the airborne irritants and microbes. Metabolic impairment in these cells has been shown to contribute to the reduced migratory ability seen in COPD patients [41], decreased phagocytosis [42] and the subsequent increase in bacterial load due to reduced clearance [43]. We have recently shown an inability of monocyte-derived macrophages from healthy smokers and COPD patients to phagocytose *S. pneumonia* and *H. influenza* [20]. Similarly, RAW 264.7 cells also showed a dose-dependent decrease in their ability to internalize S. *pneumonia* and *H. influenza* after CSC exposure (Fig. 6a, b). The decrease in the ability to phagocytose bacteria was not found to be affected by the change in the metabolic substrate for cellular respiration and energy generation. This indicates, that the defect in phagocytosis may be upstream of cellular metabolism and could be related to the non-recognition of the bacteria by the macrophage surface receptors after CS exposure. It could also be surmised that glycolysis is indispensable for bacterial phagocytosis and the metabolic shift towards fatty acid oxidation does not help in bacterial phagocytosis because of the anti-inflammatory phenotype. This could also explain the higher frequency of infections in smokers and COPD subjects.

PBMCs are increasingly being recognized for their role in characterizing the disease pathophysiology largely in inflammatory diseases. Ethical and safety constraints limit the availability of tissue samples from the site of inflammation in a number of diseases. PBMCs are thus being explored as indices/markers of diagnosis and disease monitoring due to their sensory and regulatory role in inflammation. Mitochondrial function in PBMCs is known to be affected in multiple diseases such as diabetic nephropathy [44], systemic lupus erythematous [45], schizophrenia [46], chronic fatigue syndrome [47] and sepsis [48]. PBMCs in patients suffering from sepsis have shown a very similar, generalized defect in both glycolysis and oxidative phosphorylation [48] as observed in COPD subjects here.

As the subjects in two smoke exposed groups (HS and TS-COPD) were all males and the HNS group included males and females, we tested for the possibility of gender bias and found that the results of glucose and fatty acid metabolism are not affected after adjusting for gender differences (data not shown). Most of the COPD subjects were also on therapy and no significant differences in the metabolic parameters were observed between subjects on therapy or not, in the COPD group. The mixture of six ex- and eight current smokers in the COPD group may potentially impact the results but difficult to predict given the small sample size. Previous studies have shown that the inflammatory response in smokers and ex-smokers is more or less similar [49, 50], indicating that ex-smokers also have long-lasting systemic inflammatory responses. Although these factor reflect the characteristics of COPD population in India, these could be suggested as potential limitations to our study.

## Conclusion

In conclusion, our study shows a metabolic impairment in systemic immune cell in subjects with COPD with a reduced ability to metabolize carbohydrate or fatty acids. To the best of our knowledge, this is the first study characterizing these metabolic changes in healthy smokers and COPD subjects. These observations open up new therapeutic opportunities, where specific interventions that can reverse this metabolic impairment (eg. ETC defect) may indeed improve cellular metabolism. The metabolic alterations identified here could explain the reason for frequent exacerbations in COPD subjects and also suggest that drugs that have an ability to reset the metabolic machinery may be potential novel treatment to prevent and treat COPD.

## Additional file

Additional file 1: **Figure S1.** Representative graph showing change in OCR (A) and ECAR (C) with time after addition of Glucose (10 mM, Port A), Oligomycin (4 μM, Port B), FCCP (1 μM, Port C) and 2-deoxy-glucose (50 mM, Port D). (PDF 632 kb)

## Data Availability

The datasets used and/or analysed during the current study are available from the corresponding author on reasonable request.

## Abbreviations

CS: Cigarette smoke
CSC: Cigarette Smoke Condensate
ECAR: Extracellular Acidification Rate
ETC: Electron Transport Chain
FEV_1_: Forced Expiraotry Volume in 1 s
FVC: Forced Vital Capacity
GAPDH: Glyceraldehyde-3-Phosphate Dehydrogenase
HNS: Healthy non-smokers
HS: Healthy smokers (with preserved lung function)
OCR: Oxygen consumption rate
PBMC: Peripheral Blood Mononuclear Cells
TS-COPD: Tobacco smoke related - Chronic Obstructive Pulmonary Disease
