# Supplementary figures and images for: Systemic Immuno-metabolic alterations in chronic obstructive pulmonary disease (COPD)
Source: Respir Res. 2019 Jul 30;20:171. doi: 10.1186/s12931-019-1139-2 (PMC6668083; doi:10.1186/s12931-019-1139-2)

Supplementary Fig. 1A, B

A

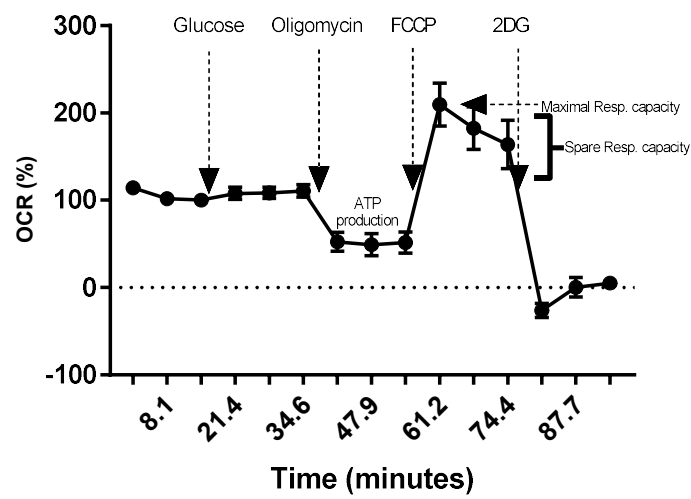

B

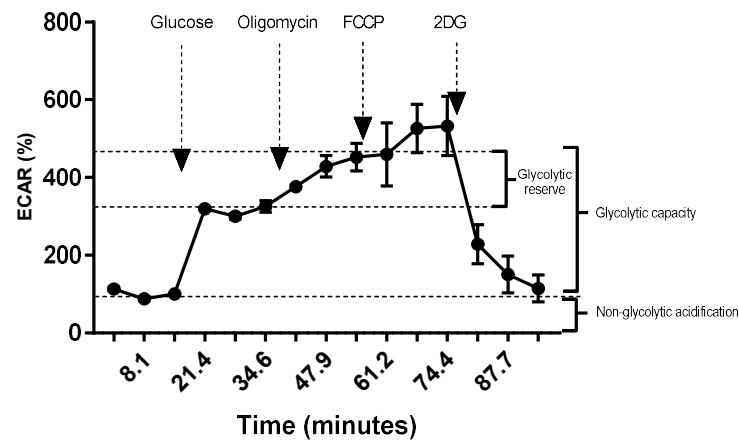

Supplement: Supplementary file 1 — Figure S1. Representative graph showing change in OCR (A) and ECAR (C) with time after addition of Glucose (10 mM, Port A), Oligomycin (4 μM, Port B), FCCP (1 μM, Port C) and 2-deoxy-glucose (50 mM, Port D). (PDF 632 kb) [file 12931_2019_1139_MOESM1_ESM.pdf]
